# Nutritional intervention is promising in alleviating liver injury during tuberculosis treatment: a review

**DOI:** 10.3389/fnut.2023.1261148

**Published:** 2023-09-21

**Authors:** Yujin Fu, Xianfa Du, Yingchun Cui, Ke Xiong, Jinyu Wang

**Affiliations:** ^1^School of Public Health, Institute of Nutrition and Health, Qingdao University, Qingdao, China; ^2^Department of Orthopedics, Affiliated Hospital of Qingdao University, Qingdao, China; ^3^Department of Infectious Diseases, The 971 Naval Hospital, Qingdao, China

**Keywords:** nutrients, functional food ingredients, tuberculosis-drug-induced liver injury, review, functional food

## Abstract

Liver injury is a main adverse effect of first-line tuberculosis drugs. Current management of tuberculosis-drug-induced liver injury (TBLI) mainly relies on withdrawing tuberculosis drugs when necessary. No effective treatment exists. Various nutrients and functional food ingredients may play a protective role in TBLI. However, a comprehensive review has not been conducted to compare the effects of these nutrients and functional food ingredients. We searched Pubmed and Web of Science databases from the earliest date of the database to March 2023. All available *in-vitro*, animal and clinical studies that examined the effects of nutritional intervention on TBLI were included. The underlying mechanism was briefly reviewed. Folic acid, quercetin, curcumin, *Lactobacillus casei*, spirulina and *Moringa oleifera* possessed moderate evidence to have a beneficial effect on alleviating TBLI mostly based on animal studies. The evidence of other nutritional interventions on TBLI was weak. Alleviating oxidative stress and apoptosis were the leading mechanisms for the beneficial effects of nutritional intervention on TBLI. In conclusion, a few nutritional interventions are promising for alleviating TBLI including folic acid, quercetin, curcumin, *L. casei*, spirulina and *M. oleifera*, the effectiveness and safety of which need further confirmation by well-designed randomized controlled trials. The mechanisms for the protective role of these nutritional interventions on TBLI warrant further study, particularly by establishing the animal model of TBLI using the tuberculosis drugs separately.

## 1. Introduction

Tuberculosis is caused by infection with *Mycobacterium tuberculosis*. According to the World Health Organization (WHO), there were 10.0 million new tuberculosis cases in 2019 (1). Standard tuberculosis treatment consists of a two-month intensive phase with isoniazid, rifampicin, pyrazinamide and ethambutol and then a four-month continuation phase with isoniazid and rifampicin. These tuberculosis drugs are known to induce liver injury and other side effects (2, 3).

During tuberculosis treatment, 5%–30% of patients exhibit varying degrees of liver injury, making tuberculosis treatment a leading cause of drug-induced liver injury (4, 5). Tuberculosis-drug-induced liver injury (TBLI) usually manifests as fever, rash, fatigue, brown urine, loss of appetite and jaundice (6). Severe liver injury can lead to liver failure and even mortality. A prospective study followed 72 patients, who were diagnosed as TBLI, and reported that 12 developed hepatic failure or hepatic encephalopathy and 9 died from these complications (7). Another study reported a similar mortality rate (67.1%, *n* = 47) among patients with TBLI (8).

Current recommendations for the management of TBLI include provider and patient education, pretreatment evaluation, regimen selection, limiting dosage and periodic clinical monitoring (3). Once hepatotoxicity occurs, the prompt withdrawal of the offending medications is the current protocol (3). However, medication withdrawal often interrupts tuberculosis treatment, hampers treatment efficacy and increases drug resistance (2).

Recent data from *in-vitro*, animal and clinical studies indicated a beneficial effect of a number of nutrients and functional food ingredients in TBLI. However, there is no consensus regarding their real effect and underlying mechanism. In this review, we first introduce the first-line drugs in standard tuberculosis treatment, their hepatotoxicity and the underlying mechanism for hepatotoxicity. Then we review the nutrients and functional foods which were reported beneficial for reducing TBLI and their potential mechanisms. The challenge and future research priorities are discussed.

## 2. Tuberculosis-drug-induced liver injury

### 2.1. Isoniazid

Hepatotoxicity occurs in about 0.10%–0.56% of patients who are treated with isoniazid alone (2, 9–12). Liver injury by isoniazid belongs to idiosyncratic hepatotoxicity, which is caused by the metabolism of the drug with little or no intrinsic toxicity (13). The main phenotype of isoniazid-induced liver injury is acute hepatocellular hepatitis (13), which is characterized by steatosis and necrosis of hepatocytes (14).

Isoniazid causes liver injury mainly because of its metabolic intermediates (14, 15). Isoniazid is converted into a variety of stable toxic substances in the body, such as acetylhydrazine, hydrazine and acetylisoniazid (16, 17). Hydrazine is the main toxic substance (15, 18, 19). Plasma hydrazine level is positively correlated with the degree of isoniazid-induced hepatic necrosis (18–20). Hydrazine is oxidized into nitrogen-containing free radicals under the action of cytochrome P450 reductase (19). This nitrogen-containing free radical is highly oxidative and can damage the cell membrane, leading to hepatocyte damage (14, 18). In addition, isoniazid metabolites can lead to mitochondria damage, resulting in the production of reactive oxygen species (ROS), which further aggravate the damage of hepatocytes (21, 22).

### 2.2. Rifampicin

The incidence of rifampicin-induced liver injury is about 2% (19, 23). The main phenotype of rifampicin-induced liver injury is cholestasis (24). Cholestasis is usually induced by bile duct injury and subsequently impaired bile flow. Cholestasis often manifests as pruritus and jaundice (25).

Bile salt outlet pump (BSEP) and Na^+^-taurocholate co-transporting polypeptide (NTCP) are both important for the transport of bile acids and the maintenance of bile acid homeostasis (25–27). Rifampicin inhibits the expression of these two bile acid transporters, resulting in the impairment of bile acid excretion (25). Rifampicin promotes the expression of bilirubin UDP-glucuronyltransferase (BGT) (28) and reduces the expression of multidrug resistance-associated protein 2 (MRP2) in hepatocytes (29). BGT is involved in the production of bilirubin (30), and MRP2 is a hepatocellular tubule transporter that plays a crucial part in bilirubin transport (29). The increased BGT and decreased MRP2 may induce hyperbilirubinemia (28, 29). The high concentration of bilirubin in the blood affects the biliary phospholipid secretion and causes canalicular membrane lesions, leading to canalicular cholestasis (31). In addition, rifampin promotes protein unfolding and misfolding, induces endoplasmic reticulum stress and aggravates cholestasis (24).

### 2.3. Pyrazinamide

Pyrazinamide can also cause liver damage. A combination of pyrazinamide and ethambutol treatment for 12 latent tuberculosis patients led to hepatic toxicity in 6 cases (19, 32). The type of pyrazinamide-induced liver injury is acute or chronic hepatitis (33). Pyrazinamide has a direct cytotoxic effect on hepatocytes and the toxicity is dose-dependent (2, 19).

Pyrazinamide was found to decrease the expression of B-cell lymphoma-2 (Bcl-2) and increase the expression of Bcl-2-associated X protein (Bax) and caspase-3, facilitating the apoptosis of rat hepatocytes (34). In addition, pyrazinamide inhibits the expression of phosphatidylinositol-3-kinase/protein kinase B signaling pathway, increases intracellular ROS and aggravates liver injury (34). Finally, pyrazinamide inhibits the nuclear factor erythroid 2-related factor 2 (NRF2)-antioxidant response element pathway, reduces the expression of downstream antioxidant proteins such as heme oxygenase-1 and sulfiredoxin 1, increases oxidative stress and induces hepatocyte damage (35).

### 2.4. Ethambutol

Currently, ethambutol is not believed to be associated with liver injury (32). The most common side effects of ethambutol are optic neuritis and vision problems (19).

## 3. Nutritional intervention for tuberculosis-drug-induced liver injury

The literature search process used the following terms: (“liver injury” OR “liver toxicity” OR “hepatoxicity”) and (“tuberculosis” or “antituberculosis” or “isoniazid” or “rifampicin” or “pyrazinamide” or “ethambutol”). The Pubmed and Web of Science databases were searched from the earliest date of the database to March 2023. During the search process, we only included the English language literature. The inclusion criteria were: (1) intervention studies (*in-vitro*, animal or human studies) which reported TBLI-related outcomes; (2) the intervention substances were commonly recognized nutrients and/or functional foods. The exclusion criteria were: (1) reviews, comments or meeting abstracts; (2) the intervention substances were combinations of common nutrients or functional foods and other substances (e.g., drugs).

As shown in Table 1, various nutrients, functional food ingredients and functional foods have been shown to be beneficial in TBLI. We graded the strength of evidence qualitatively based on the following criteria: evidence including ≥2 clinical trials or one meta-analysis rates “strong”; evidence including one clinical trial or ≥2 *in vivo* experiments rates “moderate”; evidence including one *in vivo* experiment rates “weak”; evidence including only *in vitro* experiment rates “very weak.” The ratings were also summarized in Table 1. Table 2 summarizes the potential mechanisms for the effects of various nutrients and functional food ingredients or foods on TBLI.

**Table 1:** The effects of nutritional intervention on TBLI.

Nutritional interventions	Strength of evidence^a^	Tuberculosis drug used to establish the TBLI model	Subjects (model vs. intervention)	Intervention dosage	Outcomes	The experimental type
Vitamin C (36)	Weak	Isoniazid (50 mg/kg body weight)	Male Wistar rats (10 vs. 10)	100 mg/kg body weight	AST decreased by 75%, ALT decreased by 41%, no significance on ALP	Animal
				1,000 mg/kg body weight	AST decreased by 76%, ALT decreased by 31%, no significance on ALP	
Folic acid (37, 38)	Moderate	Isoniazid (100 mg/kg body weight) and rifampin (200 mg/kg body weight)	Male SD rats (8 vs. 8)	2.5 mg/kg body weight	ALT decreased by 41%, no significance on AST and GGT	Animal
		Isoniazid	Isoniazid-induced liver injury patients (3302)		Reduced liver event reporting frequency	Human
Quercetin (39–42)	Moderate	Isoniazid (52 mmol/L)	Hep2 cells	0.1 mg/L	No significance on AST and ATL	*In-vitro*
				1 mg/L	AST decreased by 51%, ALT decreased by 59%	
		Isoniazid and rifampicin (10 mg/kg body weight)	Male SD rats (6 vs. 6)	300 mg/kg body weight	ALT decreased by 75%, AST decreased by 65%	Animal
		Isoniazid (300 mg/kg body weight)	Male SD rats (10 vs. 10)	50 mg/kg body weight	ALT decreased by 17%, AST decreased by 10%	Animal
				100 mg/kg body weight	ALT decreased by 44%, AST decreased by 38%	
		Isoniazid and rifampicin (150 mg/kg body weight)	Wistar rats (6 vs. 6)	25 mg/kg body weight	ALT decreased by 38%, AST decreased by 58%, ALP decreased by 27%, no significance on TBil	Animal
				50 mg/kg body weight	ALT decreased by 62%, AST decreased by 63%, ALP decreased by 38%, TBil decreased by 31%	
				100 mg/kg body weight	ALT decreased by 72%, AST decreased by 70%, ALP decreased by 56%, TBil decreased by 76%	
Resveratrol (43)	Weak	Isoniazid (50 mg/kg body weight) and rifampicin (100 mg/kg body weight)	BALB/c mice (6 vs. 6)	100 mg/kg body weight	AST decreased by 35%, ALT decreased by 57%	Animal
Curcumin (44, 45)	Moderate	Isoniazid (75 mg/kg body weight) and rifampicin (150 mg/kg body weight)	Male ICR mice (6 vs. 6)	200 mg/kg body weight	ALT decreased by 25%, AST decreased by 16%	Animal
		Isoniazid (100 mg/kg body weight)	Male BALB/c mice (8 vs. 8)	100 mg/kg body weight	Not available	Animal
		Isoniazid (40 mmol/L)	L-02 cells	0.5 μmol/L	ALT decreased by 13%, AST decreased by 46%	*In-vitro*
				1 μmol/L	ALT decreased by 40%, AST decreased by 27%	
				2 μmol/L	ALT decreased by 70%, AST decreased by 40%	
				5 μmol/L	ALT decreased by 87%, AST decreased by 53%	
Hesperidin (46)	Weak	Isoniazid (75 mg/kg body weight) and rifampicin (150 mg/kg body weight)	Male Wistar rat (6 vs. 6)	50 mg/kg body weight	AST decreased by 47%, ALT decreased by 69%	Animal
				100 mg/kg body weight	AST decreased by 41%, ALT decreased by 68%	
				200 mg/kg body weight	AST decreased by 49%, ALT decreased by 76%	
Chitosan (47)	Weak	Isoniazid and rifampicin (200 mg/kg body weight)	Male Wistar rats (6 vs. 6)	100 mg/kg body weight	ALT increased by 30%, AST increased by 21%, ALP decreased by 33%	Animal
*Lactobacillus casei* (48)	Moderate	Isoniazid, rifampin, pyrazinamide and ethambutol	Tuberculosis patients (105 vs. 106)	1 × 10^10^ CFU	The incidence of elevated TBil was reduced from 10 to 4%, no significance on ALP, AST, ALT and GGT	Randomized controlled trial (human)
			Tuberculosis patients (105 vs. 114)	2 × 10^10^ CFU	The incidence of elevated ALP was reduced from 5 to 0%, no significance on TBil, AST, ALT and GGT	
Spirulina (49, 50)	Moderate	Isoniazid (50 mg/kg body weight) and rifampicin (75 mg/kg body weight)	Wistar rat (6 vs. 6)	500 mg/kg body weight (administration of spirulina after isoniazid and rifampicin)	ALT decreased by 51%, AST decreased by 45%, ALP decreased by 69%, no significance on TBil	Animal
				500 mg/kg body weight (co-administration of spirulina, isoniazid and rifampicin)	TBil decreased by 55%, ALP decreased by 29%, no significance on ALT and AST	
		Isoniazid and rifampicin (50 mg/kg body weight)	Female Wistar rats (6 vs. 6)	400 mg/kg body weight	ALT decreased by 51%, AST decreased by 40%, ALP decreased by 45%, TBil decreased by 55%	Animal
				800 mg/kg body weight	ALT decreased by 46%, AST decreased by 41%, ALP decreased by 46%, TBil decreased by 58%	
Propolis (51)	Weak	Isoniazid (85 mg/kg body weight), rifampicin (65 mg/kg body weight), pyrazinamide (210 mg/kg body weight) and ethambutol (170 mg/kg body weight)	Female Wistar rats (6 vs. 6)	200 mg/kg body weight	ALT decreased by 37%, AST decreased by 38%, TBil decreased by 56%	Animal
				400 mg/kg body weight	ALT decreased by 43%, AST decreased by 39%, TBil decreased by 59%	
*Moringa oleifera* (52, 53)	Moderate	Isoniazid (7.5 mg/kg body weight), rifampicin (10 mg/kg body weight) and pyrazinamide (35 mg/kg body weight)	Male Wistar rats (6 vs. 6)	150 mg/kg body weight	AST decreased by 12%, ALT decreased by 12%, ALP decreased by 14%, TBil decreased by 17%	Animal
				200 mg/kg body weight	AST decreased by 19%, ALT decreased by 21%, ALP decreased by 29%, TBil decreased by 32%	
				250 mg/kg body weight	AST decreased by 26%, ALT decreased by 31%, ALP decreased by 46%, TBil decreased by 52%	
		Isoniazid (7.5 mg/kg body weight), rifampicin (10 mg/kg body weight) and pyrazinamide (35 mg/kg body weight)	Male Wistar rats (6 vs. 6)	150 mg/kg body weight	AST decreased by 12%, ALT decreased by 12%, no significance on ALP	Animal
				200 mg/kg body weight	AST decreased by 19%, ALT decreased by 21%, ALP decreased by 29%	
				250 mg/kg body weight	AST decreased by 26%, ALT decreased by 31%, ALP decreased by 46%	

TBLI, tuberculosis-drug-induced liver injury; AST, aspartate aminotransferase; ALT, alanine aminotransferase; ALP, alkaline phosphatase; TBil, total bilirubin; GGT, gamma-glutamyl transferase; SD rats, Sprague Dawley rats; CFU, colony-forming units.

a We graded the strength of evidence qualitatively based on the following criteria: evidence including ≥2 clinical trials or one meta-analysis rates “strong”; evidence including one clinical trial or ≥2 *in vivo* experiments rates “moderate”; evidence including one *in vivo* experiment rates “weak”; evidence including only *in vitro* experiment rates “very weak”.

**Table 2:** The mechanism of the nutritional intervention for TBLI.

Nutritional interventions	Mechanism	References
Vitamin C	Reduce oxidative stress	(36)
Folic acid	Inhibit the cannabinoid system and improve the metabolism of rifampin and isoniazid	(37)
Quercetin	Reduce oxidative stress by activating NRF2, reduce apoptosis by activating the SIRT1/ERK pathway, inhibit the NLRP3 inflammasome by regulating SIRT1 pathway and reduce inflammation by inhibiting the axis of NF-κB/TLR-4	(40–42)
Resveratrol	Reduce oxidative stress and inflammation by upregulating the expression of SIRT1 mRNA	(43)
Curcumin	Reduce the levels of protoporphyrin IX and activate the SIRT1/PGC-1α/NRF1 pathway	(44, 45)
Hesperidin	Reduce oxidative stress by up-regulating the expression of MRP2	(46)
Chitosan	Reduce oxidative stress and alter the metabolism of protein	(47)
*Lactobacillus casei*	Improve intestinal barrier function and reduce circulating LPS	(48)
Spirulina	Reduce oxidative stress	(49, 50)
Propolis	Reduce oxidative stress and inflammation	(51)
*Moringa oleifera*	Reduce oxidative stress	(52, 53)

TBLI, tuberculosis-drug-induced liver injury; NRF2, nuclear factor erythroid 2-related factor 2; SIRT1/ERK pathway, sirtuin 1/extracellular signal-regulated kinase pathway; NLRP3, nucleotide-binding oligomerization domain-like receptors family pyrin domain containing 3; SIRT1, sirtuin 1; NF-κB/TLR-4, nuclear factor kappa-B/toll like receptor 4; SIRT1/PGC-1α/NRF1 pathway, sirtuin 1/peroxisome proliferator-activated receptor-gamma coactivator 1α/nuclear respiratory factor 1 pathway; MRP2, multidrug resistance-associated protein 2; LPS, lipopolysaccharide.

### 3.1. Nutrients and functional food ingredients

#### 3.1.1. Vitamin C

Vitamin C is widely available in fruits and vegetables, especially in citrus fruits, kiwis, tomatoes and peppers (54). Previous studies suggested a protective effect of vitamin C against liver diseases, including non-alcoholic fatty liver disease (55), alcoholic liver disease (56), tetrachloromethane-(57), acetaminophen-(58) and lead-induced liver injury (59). Vitamin C is a known antioxidant, which may protect against drug-induced liver injury by scavenging toxic free radicals like ROS, inhibiting lipid peroxidation and alleviating cell membrane damage (36). In addition, vitamin C has been reported to inhibit the nuclear factor kappa-B (NF-κB) signaling pathway and reduce tumor necrosis factor-α (TNF-α), interleukin (IL)-6 and other pro-inflammatory cytokines in the liver (60). Vitamin C can also alleviate bile-acid-induced hepatocyte apoptosis by attenuating endoplasmic reticulum stress (61).

Ergul et al. (36) treated rats with isoniazid and different doses (100 mg/kg and 1,000 mg/kg body weight) of vitamin C. The results indicated that vitamin C significantly reduced the aspartate aminotransferase (AST) and alanine aminotransferase (ALT) levels in rats. In the 100 mg/kg body weight vitamin C group, AST decreased by 75% and ALT decreased by 41%. In the 1,000 mg/kg body weight vitamin C group, AST decreased by 76% and ALT decreased by 31%. The malondialdehyde level in the liver tissue was also decreased in response to the vitamin C supplementation, indicating that vitamin C may protect from isoniazid-induced liver injury via inhibiting lipid peroxidation and cell membrane damage (36).

#### 3.1.2. Folic acid

Folic acid, also termed vitamin B9, is abundant in vegetables, legumes, especially in green leafy vegetables (62). Folic acid plays a crucial role in many human biochemical processes, including the synthesis of pyrimidine and purine, and the metabolism of methionine (63). Folic acid is also in the production of S-adenosylmethionine (SAM) (64). Halsted et al. (64) indicated that reduced SAM levels could damage the nucleotide balance, which in turn led to DNA damage and induced hepatocyte apoptosis. The SAM also plays a vital role in the synthesis of glutathione which is a powerful antioxidant (64, 65).

Our recent animal experiment indicated a protective role of folic acid in TBLI (37). In the model group, rats were treated with 100 mg/kg body weight isoniazid and 200 mg/kg body weight rifampin. In the intervention group, rats were treated with 100 mg/kg body weight isoniazid, 200 mg/kg body weight rifampin and 2.5 mg/kg body weight folic acid. Compared to the model group, the ALT was decreased by 41% in the intervention group. Folic acid relieved hepatocellular necrosis, liver fibrosis and steatosis. The metabolomic analysis was conducted to explore the potential mechanism. The result indicated that folic acid changed N-acylethanolamine metabolism and therefore inhibited the cannabinoid system (37). The activated cannabinoid system is related to various liver diseases, including non-alcoholic fatty liver disease, liver cirrhosis and alcoholic fatty liver (66). Folic acid also alleviated the live inflammation by up-regulating the level of palmitoylethanolamide, and down-regulating the level of triacylglyceride and lysophosphatidylcholines (37). Additionally, folic acid accelerated the clearance of rifampin and acetylisoniazid, and therefore reduced the drug toxicity (37).

In a cross-sectional study, Suzuki et al. (38) collected 3,302 isoniazid-induced liver injury patients’ data from the WHO global individual case safety report database to identify the effects of dietary supplements on isoniazid-induced liver injury. The study reported that folic acid supplementation was associated with reduced frequencies of liver events reporting. The study concluded that folic acid may protect from isoniazid-induced liver injury.

#### 3.1.3. Phenolic compounds

##### 3.1.3.1. Quercetin

Quercetin is one of the most plentiful flavonoids in the human diet. Citrus fruits, apples, onions, coriander, berries, green tea and red wine are the main dietary sources for quercetin. Quercetin is beneficial for various diseases, including liver fibrosis, cirrhosis, cardiovascular diseases, atherosclerosis, allergic diseases and neurodegenerative diseases etc. (67, 68).

Quercetin may alleviate drug-induced liver injury by reducing oxidative stress (39). The phenolic hydroxyl groups of quercetin can react with ROS to reduce oxidative damage (21, 68). In addition, quercetin induces the expression of antioxidant enzymes like heme oxygenase-1 (67), and further eliminates ROS (69). It can also promote the activation of NRF2, which can alleviate oxidative stress in the liver (40). In addition, previous studies also indicated that quercetin can up-regulate the sirtuin 1/extracellular signal regulated kinase (SIRT1/ERK) pathway, up-regulate the expression of Bcl-2, and down-regulate the expression of cleaved caspase-3 and cleaved caspase-9 to alleviate cell apoptosis during isoniazid-induced liver injury (41, 42). Additionally, quercetin can inhibit the nucleotide-binding oligomerization domain-like receptors family pyrin domain containing 3 (NLRP3) inflammasome by regulating the SIRT1 pathway, which can alleviate liver injury (42). The activated NLRP3 inflammasome is closely associated with liver inflammation and liver injury (42). Quercetin can also decrease the inflammatory mediators (e.g., high mobility group box-1) by inhibiting the axis of NF-κB/toll like receptor 4 (TLR-4), which can alleviate liver inflammation (40).

Previous *in-vitro* and animal studies indicated a protective role of quercetin in TBLI. In a HepG2 cell experiment, isoniazid was added to cells after a one-hour pretreatment using quercetin (41). The use of 0.1 mg/L quercetin increased the cell survival rate by 57%, and 1 mg/L quercetin increased the cell survival rate by 86%. In the 1 mg/L quercetin group, ALT was reduced by 59%, and AST was reduced by 51%. Quercetin was found to significantly increase cell survival rate and decrease ALT and AST levels by regulating the SIRT1/ERK pathway (41).

In one rat experiment, the model group was treated with isoniazid, and the intervention group was treated with isoniazid and quercetin. In the 50 mg/kg body weight quercetin group, ALT was decreased by about 17% and AST was decreased by about 10% compared to the model group. In the 100 mg/kg body weight quercetin group, ALT was decreased by about 44% and AST was decreased by about 38%. Liver vacuolation, inflammatory infiltration, apoptosis and other pathological phenomena were observed in the model group, while these pathological phenomena were alleviated in the quercetin group (42). In another rat experiment, the model group was treated with isoniazid and rifampicin, and the quercetin group was treated with isoniazid, rifampicin and 300 mg/kg quercetin. Compared to the model group, ALT was decreased by 75% and AST was decreased by 65% in the quercetin group (39). Compared with the model group, the eosinophils infiltration and the parenchyma of the lobules were significantly improved in the quercetin group. In the quercetin group, the structure of hepatic lobules was intact, and the number of Kupffer cells was slightly increased (39).

In another rat experiment, the rats were treated with isoniazid, rifampicin and different doses of quercetin. In the 25 mg/kg quercetin group, ALT was decreased by 38%, AST was decreased by 58% and alkaline phosphatase (ALP) was decreased by 27% compared with the isoniazid/rifampicin group. In the 50 mg/kg quercetin group, ALT was decreased by 62%, AST was decreased by 63%, ALP was decreased by 38% and total bilirubin (TBil) was decreased by 31%. In the 100 mg/kg quercetin group, ALT was decreased by 72%, AST was decreased by 70%, ALP was decreased by 56% and TBil was decreased by 76% (40).

##### 3.1.3.2. Resveratrol

Resveratrol is commonly found in red wine, grapes and peanuts, and has certain benefits in preventing various cancers, cardiovascular diseases and neurological diseases (70). Resveratrol had a protective effect against drug-induced liver injury (71), which may be due to its anti-inflammatory and antioxidant properties. Resveratrol may reduce oxidative stress by directly scavenging free radicals or by up-regulating the activity of cellular antioxidant enzymes such as SOD, catalase and glutathione peroxidase (71). The antioxidant and free radical scavenging levels of resveratrol may also help regulate neutrophil infiltration into liver, which is shown to be involved in liver diseases (43). In addition, resveratrol inhibits NF-κB and reduces the expression of pro-inflammatory cytokines (e.g., TNF-α), which is shown to be closely related to the hepatoprotective effects of resveratrol (72). Resveratrol also up-regulates the expression of SIRT1 mRNA, which plays a protective role in liver injury (43).

Resveratrol treatment in BALB/c mice after using isoniazid and rifampicin showed that resveratrol can significantly reduce AST and ALT levels, and restore the activity of glutathione. Compared to the isoniazid/rifampicin group, resveratrol reduced AST by 35%, ALT by 57%. In the isoniazid/rifampicin group, steatosis, hepatocyte apoptosis and other pathological phenomena were observed, while these pathological phenomena were alleviated in the resveratrol group (43).

##### 3.1.3.3. Curcumin

Curcumin is a natural polyphenol that comes from turmeric (44). Previous studies suggested a protective effect of curcumin against liver diseases, including non-alcoholic fatty liver disease (73), alcoholic liver disease (74), xenobiotic-induced liver injuries such as mercury-(75) and tetrachloromethane-induced liver injury (76), which may be related to its anti-apoptosis, antioxidant and anti-inflammatory properties (77). Curcumin was found to decrease the expression of Bax, caspase-3 and phosphoinositide 3-kinase/protein kinase B signaling pathway, and reduce the apoptosis of hepatocytes (78). Curcumin can also eliminate ROS, increase the expression of SOD and glutathione, and thus decrease the oxidative damage of hepatocytes (78). In addition, curcumin can decrease the levels of protoporphyrin IX, an endogenous liver toxicant, which can alleviate isoniazid- and rifampicin-induced liver injury (44). Curcumin can also alleviate the isoniazid-induced liver injury by activating the SIRT1/peroxisome proliferator-activated receptor-gamma coactivator 1α (PGC-1α)/nuclear respiratory factor 1 (NRF1) pathway (45).

In an animal experiment by He et al. (44), mice were treated with tuberculosis drugs (isoniazid and rifampicin) with or without curcumin. Compared to the mice that were treated with isoniazid and rifampicin, the mice supplemented with 200 mg/kg body weight curcumin had a 25% lower ALT level and a 16% lower AST level. In the isoniazid/rifampicin group, the loss of hepatic architecture, centrilobular vacuolization and other pathological phenomena were observed, while these pathological phenomena were alleviated in the curcumin group (44).

Li et al. (45) treated rats with 100 mg/kg body weight isoniazid and 100 mg/kg body weight curcumin for 28 days. Curcumin alleviated the liver injury caused by isoniazid. Curcumin significantly reduced the abnormal increase of liver function biomarkers including TBil, AST, ALP and ALT. Li et al. (45). also treated the L-02 cells with 40 mmol/L isoniazid and different doses of curcumin. In the 0.5 μmol/L curcumin group, ALT decreased by 13% and AST decreased by 46% compared to the isoniazid group. In the 1 μmol/L curcumin group, ALT decreased by 40% and AST decreased by 27%. In the 2 μmol/L curcumin group, ALT decreased by 70% and AST decreased by 40%. In the 5 μmol/L curcumin group, ALT decreased by 87% and AST decreased by 53%.

#### 3.1.4. Hesperidin

Hesperidin is a flavanone glycoside that is found in large quantities in lemons and oranges. Hesperidin has antioxidant, anti-apoptosis and anti-inflammatory properties, which are vital in protecting from liver injury (79). Hesperidin can reduce oxidative stress by increasing the expression of MRP2 (46), and alleviate isoniazid- and rifampicin-induced liver injury. Hesperidin can decrease the expression of Bax and caspase-3, increase the expression of Bcl-2, and reduce the apoptosis of hepatocytes. In addition, Hesperidin can also alleviate drug- or xenobiotics-induced liver injury via its anti-inflammatory properties, which is evidenced by reduced TNF-α, IL-1β, IL-6 and NF-κB levels (80).

Zhang et al. (46) treated rats with isoniazid, rifampicin and different doses of hesperidin. In the 50 mg/kg body weight hesperidin group, AST decreased by 47% and ALT decreased by 69% compared to the isoniazid/rifampicin group. In the 100 mg/kg body weight hesperidin group, AST decreased by 41% and ALT decreased by 68%. In the 200 mg/kg body weight hesperidin group, AST decreased by 49% and ALT decreased by 76% (46). In the isoniazid/rifampicin group, portal vein inflammation, hepatocyte necrosis and other pathological phenomena were observed, while these pathological phenomena were alleviated in the hesperidin group in a dose-dependent manner (46).

#### 3.1.5. Chitosan

Chitosan is a type of marine polysaccharide that is derived from the shells of crustaceans. Chitosan has antibacterial, antioxidant, anti-liver-fibrosis and membrane stabilization properties. Chitosan promotes the expression of major antioxidant enzymes such as SOD, catalase and glutathione peroxidase (81, 82). Santhosh et al. (47) treated rats with isoniazid, rifampicin and 100 mg/kg body weight chitosan. Chitosan significantly reduced the levels of TBil, lipid peroxidation products and ALP in rats. In the chitosan group, ALT increased by 30%, AST increased by 21% and ALP decreased by 33% compared to the isoniazid/rifampicin group (47).

### 3.2. Functional foods

#### 3.2.1. Probiotics

Probiotics are health-promoting living microorganisms for the host. Several probiotics were shown to play a beneficial role in liver diseases including non-alcoholic fatty liver disease, cirrhosis and drug-induced liver injury (83–86). Probiotics may help improve intestinal barrier function, reduce circulating lipopolysaccharide (LPS) in the body and eventually reduce drug-induced liver injury (48, 87). Elevated LPS may induce liver injury through several mechanisms, including inducing massive accumulation of bile acids and overproduction of ROS, which promote the apoptosis and necrosis of hepatocytes (88–90).

Our recent randomized controlled trial (48) indicated that *Lactobacillus casei* supplementation alleviated the abnormal increase of cholestasis-related liver indices during tuberculosis treatment. In the control group, tuberculosis patients received a standard therapy which consisted of a two-month intensive phase using isoniazid, rifampin, pyrazinamide and ethambutol, followed by a four-month continuation phase using isoniazid and rifampin. In the intervention group, tuberculosis patients received the standard therapy plus an additional 1 × 10^10^ colony-forming units (CFU) per day (low-dose group), or 2 × 10^10^ CFU per day of *L. casei* (high-dose group). The *L. casei* supplementation lasted for 2 months. The incidence of elevated ALP was reduced from 5% to 0% in the high-dose group vs. the control; while the incidence of elevated TBil was reduced from 10% to 4% in the low-dose group vs. the control. These changes were associated with a significant reduction in LPS, a significant improvement in intestinal barrier function and a reshaping of the gut microbiota.

#### 3.2.2. Spirulina

Spirulina is the commercial name for cyanobacteria that live in fresh water and alkaline salt water (91, 92). Spirulina is a frequently consumed vegetable and has been widely utilized in food products such as snacks, pasta and sweets (92). Spirulina mainly refers to *Spirulina maxima* and *Spirulina fusiformis* (92). Spirulina has a high protein content and contains a variety of nutrients and functional ingredients, such as phycocyanin, γ-linolenic acid, vitamins and β-carotene etc. (49, 93). Phycocyanin exerts anti-inflammation activity by inhibiting the expressions of TNF-α, IL-6 and IL-1 (93). β-carotene exerts anti-inflammation activity by inhibiting the NF-κB pathway (93, 94). Spirulina species has attracted increasing attention as a nutritional supplement due to its anti-oxidation properties. *S. fusiformis* and *S. maxima* play a crucial role in protecting from drug-induced liver injury (49, 50).

In an animal study, rats were treated with 500 mg/kg body weight *S. maxima* for 2 weeks after isoniazid- and rifampicin-induced liver injury. Compared with the isoniazid/rifampicin group (left untreated), *S. maxima* decreased ALT by 51%, AST by 45%, ALP by 69%. In another experiment in the same study, rats were co-administrated with *S. maxima* (500 mg/kg body weight), isoniazid and rifampicin for 4 weeks. Compared with the isoniazid/rifampicin group, *S. maxima* decreased TBil by 55% and ALP by 29%. At the same time, *S. maxima* restored oxidative stress biomarkers such as SOD and catalase to a normal level (49).

In another rat study, isoniazid and rifampicin treatment significantly increased the average liver weight. *S. fusiformis* (400 or 800 mg/kg body weight) significantly alleviated the abnormal increase of liver weight. In addition, *S. fusiformis* significantly reduced the abnormal increase of liver function biomarkers including TBil, AST, ALP, ALT and restored the activities of antioxidant enzymes including SOD, catalase, glutathione etc. In the 400 mg/kg body weight *S. fusiformis* group, AST decreased by 40%, ALT decreased by 51%, ALP decreased by 45% and TBil decreased by 55% compared to the isoniazid/rifampicin group. In the 800 mg/kg body weight *S. fusiformis* group, AST decreased by 41%, ALT decreased by 46%, ALP decreased by 46% and TBil decreased by 58% compared to the isoniazid/rifampicin group. In addition, *S. fusiformis* alleviated the liver periportal inflammation and the congestion of central vein compared with the isoniazid/rifampicin group (50).

#### 3.2.3. Propolis

Propolis is a resin material that bees collect from different kinds of plants (95). Propolis has been utilized in food and drinks for a long time in human history (91, 96). There are a variety of functional food ingredients in propolis, mainly including phenolic acids and flavonoids, which can play a protective role in liver by scavenging free radicals (95). Caffeic acid phenethyl ester is a phenolic compound in propolis. Caffeic acid phenethyl ester can induce the kelch-like ECH-associated protein 1/NRF2/antioxidant response element pathway, increase the expression of the heme oxygenase-1 and thus exert antioxidant effects (97). Caffeic acid phenethyl ester also has anti-inflammatory properties by suppressing the activation of NF-κB (98). Propolis may relieve TBLI by reducing oxidative stress and inflammation (95).

Bhadauria treated female rats with isoniazid, rifampicin, pyrazinamide, ethambutol and different doses of propolis. Propolis significantly reduced ALT, AST and TBil levels in rats. In the 200 mg/kg body weight propolis group, ALT decreased by 37%, AST decreased by 38% and TBil decreased by 56% compared with the isoniazid/rifampicin/pyrazinamide/ethambutol group. In the 400 mg/kg body weight propolis group, ALT decreased by 43%, AST decreased by 39% and TBil decreased by 59%. Compared with the isoniazid/rifampicin/pyrazinamide/ethambutol group, liver cell necrosis, lymphocyte infiltration, vacuolation and other liver-injury-related pathological phenomena were reduced after propolis treatment (51).

#### 3.2.4. Moringa oleifera

*M. oleifera* is an edible vegetable that is mainly consumed in tropical and sub-tropical countries (99, 100). *M. oleifera* leaves as a food fortifier play an important role in food products such as bread, cereal porridge and yoghurts (101). *M. oleifera* seeds as cooking oil raw materials have spread from India to other parts of the world (101). *M. oleifera* contains a variety of nutrients and functional food ingredients, such as vitamin A, flavonoids and β-carotene (102). *M. oleifera* may alleviate TBLI by reducing oxidative stress via directly scavenging free radicals (52) or increasing the expression of the glutathione peroxidase, and glutathione reductase (102). In addition, *M. oleifera* exerts anti-inflammation effects by suppressing the expression of the isoform of nitric oxide synthase, cyclooxygenase-2 and NF-κB signaling pathway (102), and seems to keep the structural integrity of liver cell membranes (53).

Pari et al. (53) treated rats with isoniazid, rifampicin, pyrazinamide and different doses of *M. oleifera* leaf extract (MLE). MLE significantly reduced ALP, TBil, AST and ALT levels in a dose-dependent manner. In the 150 mg/kg body weight MLE group, AST decreased by 12%, ALT decreased by 12%, ALP decreased by 14% and TBil decreased by 17% compared with the isoniazid/rifampicin/pyrazinamide group. In the 200 mg/kg body weight MLE group, AST decreased by 19%, ALT decreased by 21%, ALP decreased by 29% and TBil decreased by 32%. In the 250 mg/kg body weight MLE group, AST decreased by 26%, ALT decreased by 31%, ALP decreased by 46% and TBil decreased by 52% (53). In addition, in the MLE treatment group, hepatic sinus dilatation, portal inflammation, microvesicular steatosis and other liver-injury-related pathological changes were significantly reduced (53).

In another study by Ashok Kumar et al. (52), rats were treated with isoniazid, rifampicin, pyrazinamide and different doses of MLE. MLE significantly reduced AST, ALT and ALP levels in a dose-dependent manner. In the 150 mg/kg body weight MLE group, AST decreased by 12% and ALT decreased by 12% compared with the isoniazid/rifampicin/pyrazinamide group. In the 200 mg/kg body weight MLE group, AST decreased by 19%, ALT by 21% and ALP by 29%. In the 250 mg/kg body weight MLE group, AST decreased by 26%, ALT by 31% and ALP by 46% (52).

## 4. Conclusions and future directions

Based on comparing the strength of the evidence, the evidence for the protective effect of folic acid, quercetin, curcumin, *L. casei*, spirulina and *M. oleifera* on TBLI was moderate. The other substances were rated “weak” because limited studies were available. Most current evidence for the protective role of nutrients and functional food ingredients/foods on TBLI was from animal studies. Well-designed randomized controlled trials need to be conducted to further the effectiveness of these substances. Furthermore, the adverse effects of the functional food ingredients/foods were not understood and warrant future investigation.

Alleviating oxidative stress and apoptosis were the leading proposed mechanisms for the beneficial effects of the nutrients and functional food ingredients/foods on TBLI. Two or more tuberculosis drugs were usually used to establish the animal models in these studies. Rifampicin typically leads to cholestasis-type liver injury (24), while isoniazid and pyrazinamide lead to hepatitis-type liver injury (13, 33). Future work may establish the animal models of TBLI by each tuberculosis drug separately to better illustrate the mechanisms of these nutrients and functional foods on tuberculosis-drug-induced liver injury. Also, the protocols to establish the animal models of TBLI (e.g., animal type, drug dosage and duration) may be standardized to allow comparisons between studies.

Epidemiological studies indicated low body-mass index (BMI) as a crucial risk factor for TBLI (103, 104). Malnutrition, including low BMI and deficiency of protein and multiple vitamins is one of the features of tuberculosis patients (105–107). Protein deficiency is known to affect drug metabolism and may play a role in TBLI. The relationship between protein, micronutrients deficiency and the incidence of TBLI was rarely explored and may be a future research direction. Our recent cohort study (108) indicated that a high vegetable intake and a low cooking oil intake were associated with a reduced risk of TBLI. An optimal dietary pattern may be further investigated for reducing the risk of TBLI.

In conclusion, a few nutritional interventions including folic acid, quercetin, curcumin, *L. casei*, spirulina and *M. oleifera* showed promise in alleviating TBLI, the effectiveness and safety of which need further confirmation by well-design randomized controlled trials. The mechanism of the protective effects of these substances needs further research, particularly by establishing animal models of TBLI using tuberculosis drugs separately. This work provides the basis for future recommendations for nutritional intervention on TBLI.

## Author contributions

YF: Writing – review & editing, Investigation, Writing – original draft. XD: Writing – review & editing, Investigation. YC: Writing – review & editing, Investigation. KX: Writing – review & editing, Funding acquisition, Methodology. JW: Funding acquisition, Methodology, Writing – review & editing, Conceptualization, Project administration.

## Funding

The author(s) declare financial support was received for the research, authorship, and/or publication of this article. This research was funded by the National Natural Science Foundation of China (Nos. 82003446 and 82103847); Young Elite Scientists Sponsorship Program by China Association for Science and Technology (No. 2020QNRC001).

## Conflict of interest

The authors declare that the research was conducted in the absence of any commercial or financial relationships that could be construed as a potential conflict of interest.

## Publisher’s note

All claims expressed in this article are solely those of the authors and do not necessarily represent those of their affiliated organizations, or those of the publisher, the editors and the reviewers. Any product that may be evaluated in this article, or claim that may be made by its manufacturer, is not guaranteed or endorsed by the publisher.
